# Preschool Healthy Food Policy Did Not Increase Percent of Food Wasted: Evidence from the Carolinas

**DOI:** 10.3390/nu12103024

**Published:** 2020-10-02

**Authors:** Roni A. Neff, Daniel A. Zaltz, Amelie A. Hecht, Russell R. Pate, Brian Neelon, Jennifer R. O’Neill, Sara E. Benjamin-Neelon

**Affiliations:** 1 Department of Environmental Health & Engineering, Johns Hopkins Bloomberg School of Public Health, 615 N Wolfe St, Baltimore, MD 21205, USA; 2 Center for a Livable Future, Johns Hopkins Bloomberg School of Public Health, 615 N Wolfe St, Baltimore, MD 21205, USA; 3 Department of Health, Behavior and Society, Johns Hopkins Bloomberg School of Public Health, 615 N Wolfe St, Baltimore, MD 21205, USA; dzaltz1@jhu.edu (D.A.Z.); sara.neelon@jhu.edu (S.E.B.-N.); 4 Department of Health Policy and Management, Johns Hopkins Bloomberg School of Public Health, 615 N Wolfe St, Baltimore, MD 21205, USA; ahecht3@jhu.edu; 5 Department of Exercise Science, University of South Carolina Arnold School of Public Health, 921 Assembly St, Columbia, SC 29208, USA; RPATE@mailbox.sc.edu (R.R.P.); ONEILLJR@mailbox.sc.edu (J.R.O.); 6 Division of Biostatistics, Department of Public Health Sciences, Medical University of South Carolina, 35 Cannon St, Charleston, SC 29415, USA; neelon@musc.edu

**Keywords:** food waste, early care and education, school nutrition standards, preschool, evaluation, policy

## Abstract

This research evaluates the effects of a South Carolina (SC) policy, which changed the nutrition standards for foods served in early care and education (ECE) settings, on wasted food. A two-group pre-test/post-test evaluation was performed in ECE centers serving children age 3–5 from households with lower incomes in SC (*n* = 102 children from 34 centers, intervention) and North Carolina (NC; *n* = 99 children from 30 centers, comparison). Direct observation was performed to assess the quantity and kcal of food served and quantity and percent of food discarded, by food group and nutrient, enabling assessment of waste in the absence of intervention. Mixed-effects linear models were fit to estimate, by state, differences in change from baseline to post-implementation at the center level. Covariates were selected a priori, including center enrollment, racial composition, director educational attainment, years in operation, for-profit status, and Child and Adult Care Food Program (CACFP) participation. Waste of food was high across states and time points. The policy was not associated with a change in percent of food discarded in SC compared to NC in adjusted analyses.

## 1. Introduction

An estimated 40% of the US food supply is wasted, with considerable effects on the environment, budgets and public health [1,2]. By one estimate, consumers and consumer-facing businesses and organizations may be responsible for over 80% of the country’s waste of food [2]. Several studies have found that waste of food is especially high among young children and in households with children, although the literature is mixed [3,4,5]. Young children are prone to neophobia (fear of the new), and some evidence suggests a greater biological preference for foods that are sweet, salty and high in fat among young children compared to older age groups [6,7]. Accordingly, young children may reject foods served to them that do not fall into these categories or that are less familiar. Another reason for high waste of food among young children is that challenges in communicating about or recognizing their hunger and satiety levels may lead to being served more food than is wanted.

Approximately 60% of US children under five years old are in regular child care arrangements, including early care and education (ECE) programs [8]. The term ECE refers to child care services by non-parents outside the family home, including center-based and home-based child care. Here, we focus on center-based services for children aged 3–5 years old. These services commonly provide one or more meals and snacks, some funded by parent fees and others by governmental programs. Numerous healthy eating policies and interventions have been developed for ECE programs, in part because evidence suggests lifelong food consumption patterns and attitudes may be initiated in early childhood [7].

Plate waste is often used to assess impacts of healthy foods initiatives, representing the inverse of consumption. High plate waste among foods targeted in nutrition interventions is often seen as a reflection of unsuccessful intervention implementation, but fewer studies place primary focus on the waste in itself. Focusing on waste and its antecedents is important, not only because it reflects loss of funds for ECE programs, which typically operate on tight budgets, but also because children ultimately benefit when programs jointly build healthy habits in both nutrition and food wastage.

The vast majority of school nutrition research using plate waste as an outcome has been performed among older children in elementary schools and above, with multiple studies in particular assessing the impact of revised nutrition standards mandated by the Healthy, Hunger-Free Kids Act of 2010 [9]. While media reports and concerns expressed by school foodservice staff suggested that the federal regulations resulted in increased plate waste, research suggests otherwise. For example, a multi-year cross-sectional study of school meals found no impact on plate waste, while a nationally-representative survey of school staff found the lunches were accepted [10,11]. A 2017 review found that in four of five regional studies, there was no increase in waste of fruits and vegetables [12]. A longitudinal study in urban middle schools found that waste of entrees and vegetables was reduced following the policy [13]. Further, studies showed the regulations were associated with improved diet quality [14,15].

We identified three studies examining plate waste in ECE in conjunction with healthy food initiatives, none finding increased waste. Esquivel et al. (2016) assessed the impact of a pilot obesity prevention policy intervention in Hawaii Head Start centers. The intervention included increased fruits, eliminating juice and offering family-style meals, monthly lessons on fruits and vegetables, and teacher training [16]. Post-intervention, children discarded a reduced percentage of the fruits served, with no change in vegetable discards. (The study reported consumption, but the measurement involved plate waste.) Woodward-Lopez et al. (2018) examined shifts in consumption following family child care participation in a multi-sector community-wide obesity prevention initiative in Northern California [17]. The providers received training and they individually selected practices to adopt, including changes to feeding, mealtime environment, and parent engagement. The study found that although lunch healthfulness served improved modestly, there was no significant change in plate waste for entrees, fruits, vegetables, grains/starches or beverages. Lastly, Seward et al. (2017) evaluated a provider-focused intervention addressing compliance with child care nutrition standards in New South Wales, Australia. The intervention included executive support, staff training and informational resources, audit and feedback, and technical assistance. The intervention increased compliance, with no change in percentage of food wasted, after adjusting for baseline differences [18].

These three interventions all included behavioral components, whether for the child or the provider. We have not identified studies examining ECE plate waste in response to changed nutrition standards only, though as described above, some literature assesses changes in nutrition standards among older children. It is useful to evaluate nutrition standards as an intervention because they may be more sustainable and less costly than behavioral interventions.

The present study seeks to understand the effects of one such policy. In 2012, South Carolina (SC) implemented a set of 13 mandatory nutrition standards (Table 1) in its ABC Quality program, a statewide subsidized child care program for families earning less than 150% of the federal poverty level [19]. The program complements the national Child and Adult Care Food Program (CACFP), which subsidizes food and beverages within ECE programs based on need, so long as the food meets nutritional standards. An evaluation of compliance with the SC standard found small gains in healthy food provision, but a need for further efforts to increase compliance [20]. In addition to cost, the other top barrier to compliance was a provider view that children would dislike the new menu. An evaluation of policy impact found improved consumption of fruits and lean protein, though dairy consumption decreased and overall diet quality did not improve as intended [21]. Despite this outcome, we refer to the policy as a healthy foods policy, given its intent and the observed increases in serving healthy foods.

This study takes a difference-in-difference approach to evaluating food waste among children aged 3–5 in an ECE setting in SC before and after the policy, compared to the same time period in North Carolina (NC), a bordering state that did not implement a contemporaneous child nutrition policy. We hypothesized that the percent of served food that was discarded would not increase post-intervention in SC ECE centers compared to NC centers. This research also contributes to understanding food wastage patterns in young children more broadly, by characterizing the foods most discarded in the study ECE centers in the absence of intervention.

## 2. Materials and Methods 

### 2.1. Data Collection

The data collection methods and sample have been described in detail elsewhere [20] and are briefly summarized here. The study included 34 ECE centers serving families with lower incomes in the Columbia, SC area, and 30 in the Raleigh, NC area.

Using the Diet Observation in Child Care (DOCC) [22] approach, trained data collectors visited each center before and after SC policy implementation, observing three children from one randomly selected classroom per center during one regular full day of care. Data collectors arrived prior to opening and stayed until the last child had left the center. They tracked all foods served, traded, discarded and remaining, for all meals and snacks. Data collectors received extensive training in use of the DOCC to visually assess food and beverage portions and were blinded to study aims. Pre-implementation data were collected for 102 children in SC and 90 in NC; and 9 months later, post-implementation data were collected for 99 children in SC and 78 in NC. Follow-up data was missing from four centers (three in NC and one in SC), with three centers having closed and the fourth declining to participate. Centers lost to follow-up were demographically similar to those that participated at both timepoints. Because no identifying information was collected about the children, the pre- and post-implementation samples did not necessarily include the same children.

Center directors also responded to questionnaires regarding center characteristics. As was previously documented, centers were similar across states on most criteria. However, compared to centers in NC, centers included at baseline in SC were on average larger (46 enrolled vs. 25), had a lower percentage of Hispanic children (1.4% vs. 4.8%), were less frequently for-profit (18% vs. 26%) and directors were more highly educated (39% more than 4-year college vs 7%).

The Institutional Review Boards of the University of South Carolina (#00014606) and Duke University Medical Center (#00033793) approved this study, both in 2012.

### 2.2. Analysis

Nutritional composition of the diet data was analyzed using the Nutrition Data System for Research 2012 (NDSR) [23]. Findings were calculated per day at the center level, averaging across the three children in each center for each food, beverage, micronutrient and macronutrient. Waste was estimated both as the percentage of served that was not consumed, and the absolute difference between served and consumed. In a few instances, the quantity of food, beverage or nutrient recorded as consumed for a child exceeded the amount served to that child. These errors, likely attributable to non-recorded second helpings or food trades with peers, occurred in observations of 8 children across 5 centers (2% of observations). In these instances, we set served equal to consumed for the specific item for that child.

Means, standard deviations (SD), and proportions were calculated to summarize center characteristics. We subtracted consumed from served to derive estimates for quantities wasted.

To evaluate policy impact, unadjusted and adjusted mixed effects linear models were fit to estimate the difference, by state, in the change from baseline to post-policy implementation (difference-in-difference) in foods, beverages, micronutrients and macronutrients served and wasted (total quantity and as a percent of served) [24]. All models included center random effects and indicators for time, state, and an interaction between time and state (difference-in-difference estimator). Adjusted analysis controlled for the following fixed effect covariates, selected a priori [21]: number of three- to five-year-olds enrolled, proportion of children who are Black >50%, director educational attainment (more than high school or community college vs. some or all of 4-year college or graduate degree), years center has been in operation, profit status (for-profit vs. nonprofit), CACFP participation (yes vs. no). We used robust standard errors to account for clustering at the center level. Results are reported as predicted probabilities and marginal effects.

Analyses were conducted in Stata/IC version 14.1 (StataCorp LP, College Station, TX, USA) with a significance level of *p* < 0.05.

### 2.3. Outcomes Reported

We first describe the foods discarded in the highest percentage in the absence of the policy (in SC before intervention and at both time points in NC). This information serves both to ground the policy impact analysis, and also to provide information useful for understanding food waste among young children in general. Next, we report on difference-in-difference in the amounts of food served between states before and after the policy, and finally, the difference-in-difference in the amounts discarded.

## 3. Results

**Figure 1:**
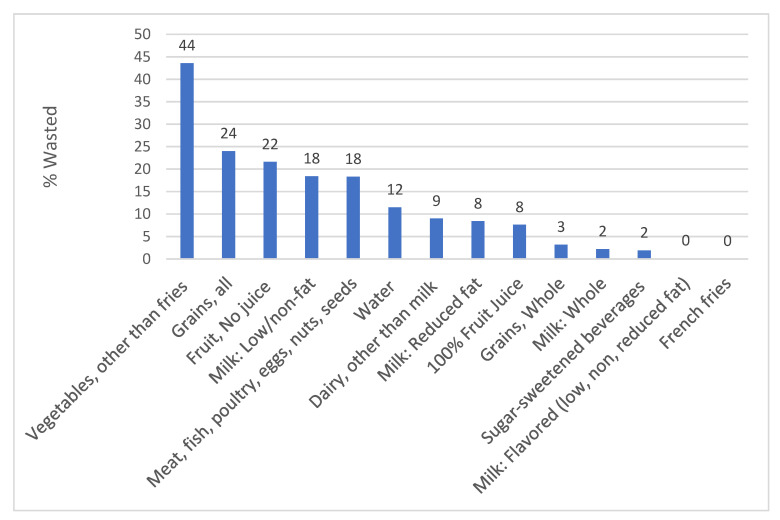
Percent of selected foods and beverages wasted in the absence of the policy, in reference to total amount served (North Carolina pre-and post-intervention plus South Carolina pre-intervention) (*n* = 90 observations).

Figure 1 summarizes the most wasted items among the young children in the absence of the nutrition policy (pre-intervention SC combined with both time-points in NC). The category wasted in the greatest percentage (mean, SD) was vegetables not including fries (44%, SD = 28%). The next most wasted food categories were all beverages (22%, SD = 18%); fruit not including juice (22%, SD = 22%), and milk (21%, SD = 18%). Items with less than 3% waste included flavored milks, fries, sugar-sweetened beverages, and non-dairy milks. Because vegetables are high in nutrients, we found that preschoolers discarded 32% (SD = 23%) and 30% (SD = 20%), respectively, of the amount of vitamins A and C served to them.

The policy’s potential effect on food wastage results, in essence, from serving food in excess of willingness to consume. Accordingly, it is important to know how the policy affected the quantities of food served. Table 2 characterizes the shift in foods and beverages served from baseline to follow-up in the two states. Results were similar in direction, magnitude, and statistical significance in unadjusted and adjusted models; results discussed in the text below are from adjusted models. In the post-intervention period, compared to sites in NC, SC sites significantly increased their serving of vegetable protein, fruits not including juice, and grains; as well as calories and carbohydrates, after adjusting for covariates. For several other food groups and nutrients, significant changes were observed between the intervention or comparison groups across time, but the comparison of changes *between* the two states was not significant.

We next examined change over time in the percentage of served food that was wasted across the two states, controlling for the covariates (Table 3). Despite the serving changes observed in some categories, there were virtually no differences between the states in how discards changed over time. The one exception was milk: the intervention group discarded 6% more post-intervention, while milk discards were actually reduced by nearly 7% in the comparison group (difference-in-difference was 12.6%, *p* = 0.01). Supplementary Table S1 provides further context on the increased milk discards, indicating a shift in types of milk served before and after the policy. Initially, 59% of SC centers served whole and reduced fat (2%) milk (versus low fat (1%) and nonfat milk), while post-policy, that percentage dropped to 39%.

## 4. Discussion

The studied preschoolers wasted high portions of food served to them. In schools not affected by the SC policy (NC and SC pre-intervention), they discarded 44% of vegetables (excluding fries) and over 20% each of the fruit and milk served. The SC policy resulted in increased serving of vegetable protein, fruit, grains, calories and carbohydrates, but no resultant increase in waste compared to NC. Increased waste might have been expected in SC given evidence that serving larger portions increases both consumption and total waste, a finding that has been replicated in preschoolers [25,26].

As described in [21], the SC policy was not found to result in improved nutrition, as measured by the Healthy Eating Index, although it did increase produce consumption. Correspondingly, we document increased serving of healthy products including fruits and vegetables. Despite young children’s frequent preference for sugar, salt and fat, we found that the increased serving did not result in produce waste.

The policy did, as intended, result in increased servings of several foods, so that although the percentage discarded was stable, the absolute amounts discarded increased for calories, protein, vegetables, grains and sugar in SC. Nonetheless, because the amounts served and discarded also increased in NC, the difference-in-difference findings were not significant. Further research is needed to understand this finding.

Milk was the only food item that showed a differential increase in percent waste. This change was likely due to a shift toward low or nonfat milk with the standards. The two prior ECE plate waste studies presenting milk and beverage data likely also found increased milk waste, though the finding was not directly reported. Both reported no significant changes in consumption despite increased dairy serving [18] and compliance with milk guidelines [17]. Over time, as children adjust their taste preferences for low and nonfat milk, it is possible that consumption may increase and waste decrease. In the meantime, it may be helpful to reduce quantities of milk served, if allowed by the policy.

Reactions to new school meal regulations can be expected to differ between the young children from lower income backgrounds observed in this study versus older children or those from higher resource households. The differences could, however, go in either direction. For example, compared to older children, young children might have elevated waste due to stronger neophobia and preferences for sugar, fat, and salt [6,7]. Yet, protective factors include relatively little memory of the foods served under a prior regime and a lack of complementary options to obtain alternative foods beyond what is served. Children from households with lower incomes may be especially likely to eat what is served at school because there may be more limited supplementary food offered at home; or alternately, may be more likely to waste if they have reduced familiarity with fresh produce items due to barriers to serving them at home [27,28].

ECE settings provide a greater opportunity for providers to influence children’s behavior than do primary schools, due to the generally lower child-to-provider ratio. Evidence suggests, however, that many providers use this influence to encourage finishing plated food [29,30,31]. Although the motivation behind such pressure may be waste prevention, overriding satiety cues may ultimately increase waste, leading to unnecessary consumption (a form of waste), and contributing to a continued pattern of excess serving. This research did not include the study of provider behavior and the intervention did not include work with providers. Provider interventions represent an important future direction for ECE food waste reduction efforts and evaluation.

Plate waste methodology is widely used to provide a proxy for consumption, but school nutrition studies rarely focus on the data’s direct utility. This analysis highlights the value of using these data to gain insight about waste of food, and also of studying food consumption and food waste jointly. The high burden of wasted food in the US and globally incurs large, unnecessary costs. In the case of ECE programs, ongoing discards of such a high percentage of food mean unnecessary expenditure of program dollars that could have been used for needs such as feeding children higher quality foods and paying staff higher wages. We found that vegetables are wasted at particularly high rates, signifying a particularly high loss of nutritional and economic value [32]. Further, the food that is later discarded could have been providing nourishment to children. Overnutrition and waste share risk factors including portion size, cultural attitudes toward food valuation, and pressures for adults to be “good providers ”[33,34].

There are many opportunities to create synergies between efforts to reduce waste of food and improve nutrition; however, depending on how policies and interventions are implemented, it is also possible that the two goals will compete [33]. No identified resource focuses on addressing waste of food at the preschool level; however, it is possible to adapt recommendations from some of the excellent practical resources and guides targeted to the kindergarten–grade 12 (primary and secondary education) school level (examples include [35,36].) The “waste” discussion should be expanded to weigh the relative utility in discarding food in some contexts. One example is in encouraging children to discard unwanted foods (but work on better assessing how much to take in the first place), versus training children to clean their platters no matter what. Additionally, if providing excesses or greater variety of healthy foods increases consumption, then there can be benefit even if children do not consume the entire surplus. The studied policy, which successfully increased portions of some healthier foods without increasing waste, may contribute to the ability to identify portion thresholds for optimizing both goals.

This represents one of the first studies to focus on waste as an outcome in preschoolers. It is based on a rigorous observational method of data collection, including specific information on what was served, compliance with the policy, and actual discards. The approach further includes pre/post observations in the intervention and comparison states, and a rigorous method of nutrient analysis. However, the study had a relatively small sample size, and ECE facilities differed in some ways across states. Unexplained and often similar-direction changes occurred in waste in NC. This secular change may have contributed to the finding that the difference-in-difference for direct waste quantities was not statistically significant. Another unexplained finding was that the policy led to increased calories served. Additionally, it is possible that behaviors in the pre-intervention time period, nine months before policy implementation, were affected by news about a potential future policy change. Lastly, the study did not collect information about actual provider communication or actions, so we do not have information to understand the experiences children may have had in their centers and during the studied meals.

Research is needed across additional settings to add to the evidence base and provide further insights into waste of food by preschoolers and how it may be affected by healthy food policies and other interventions. Further research is warranted to assess the role of serving size in waste and child nutrition. It would also be helpful to assess whether rough balance points could be identified beyond which increased serving is especially likely to go unconsumed, by food item, age group and other factors. Additionally, documenting what foods are most wasted can suggest needs to either adapt menus and recipes, or intervene to increase demand for the identified foods. Further, understanding determinants of waste, including waste as a function of child and center-level characteristics, may point to additional systems-level interventions to improve nutrition and reduce waste of food.

## 5. Conclusions

This research suggests that the ECE healthy food policy in SC was successful in increasing healthy food consumption without increasing overall waste. This study also highlights the foods most likely to be wasted in ECE. The study contributes to a small literature focused on the waste of food in young children and ways to address it. The waste of food carries high costs for feeding programs and households, among its damaging effects. There is a great need for more research to establish best practices for food waste reduction in ECE and other settings where young children spend time. Embedding this food waste study within a larger policy evaluation study highlights the potential tradeoffs, and the need for a systems perspective focusing on optimizing benefits across nutritional and food waste outcomes.

## Figures and Tables

**Table 1:** ABC Quality Program nutrition standards [19].

Beverages	Skim or 1% milk for children 2 y and older
	No sugar-sweetened beverages
	Juice once per day or less, 4-oz servings
Fruits and vegetables	At least 2 different fruits served 2 or more times per day
	Vegetable other than white potatoes at least 1 time per day
	Fried or prefried vegetables served 1 time per week or less
Whole grains	Whole-grain foods served once per day
Other foods	High-fat meats served 2 times per week or less
	Sweet food items served 2 times per week or less
Policies and practices	Staff attend nutrition training at least 1 time per year
	Children learn about nutrition at least 1 time per week
	Do not use food as a reward or punishment
	Create and consistently implement a written nutrition policy

ABC is not an acronym; the letters, A,B, and C correspond to quality ratings.

**Table 2:** Change in quantity of food, beverages, and macronutrients served per child per day by study arm, (*n* = 34 centers, averaging across included children).

		Unadjusted ^a^				Adjusted ^a^	
		Baseline	Follow-Up	Diff. from Baseline	*p*-Value ^b^	Diff. from Baseline	*p*-Value ^b^
Total food, beverages served (g)	Intervention						
	Comparison	971.99	1094.10	122.10	0.002 **	124.22	0.003 **
	Difference	926.58	966.92	40.33	0.36	37.32	0.39
				81.77	0.17	86.91	0.15
Fruit, No juice (cups)	Intervention	0.48	0.63	0.15	0.01 **	0.15	0.01 **
	Comparison	0.65	0.54	−0.11	0.13	−0.12	0.11
	Difference			0.26	0.00 **	0.27	0.004 **
Vegetables, No fries (cups)	Intervention	0.44	0.65	0.22	0.00 **	0.20	0.00 **
	Comparison	0.41	0.48	0.07	0.30	0.07	0.32
	Difference			0.15	0.11	0.13	0.16
Grains, All (cups)	Intervention	1.26	1.79	0.53	0.00 **	0.56	0.00 **
	Comparison	1.47	1.60	0.13	0.25	0.13	0.26
	Difference			0.40	0.01 **	0.43	0.01 **
Beverages, Milk (fluid oz)	Intervention	15.08	14.35	−0.73	0.41	−0.34	0.70
	Comparison	12.02	14.02	2.00	0.02 *	1.93	0.02 *
	Difference			−2.73	0.02 *	−2.27	0.06

^a^ Results are from mixed-effects linear regression models. Adjusted models control for ethnicity (>50% black students), number of 3, 4 and 5-year olds enrolled, director education level (HS/Community College vs. Some/All 4-yr College/Graduate Degree), the number of years the center has been in operation, participation in the Child and Adult Care Food Program, and the center’s profit status (for-profit vs. nonprofit). All models (adjusted and unadjusted) include center random effects and robust standard errors. Results are reported as predicted probabilities and marginal effects. Percentages of food, beverages and nutrients are presented at the center-level, averaging across the three included children per center. ^b^ *p*-values are for change from baseline within study arm or difference-in-difference across study arms. * Significant at the *p* = 0.05 level; ** Significant at the *p* = 0.01 level.

**Table 3:** Change in percent wasted per child per day by study arm (*n* = 64 centers, averaging across included children).

		Unadjusted ^a^				Adjusted ^a^	
		Baseline	Follow-Up	Diff. from Baseline	*p*-Value ^b^	Diff. from Baseline	*p*-Value ^b^
Total food, beverages wasted (g)	Intervention	27	29	2	0.56	3	0.33
	Comparison	22	23	1	0.80	1	0.82
	Difference			1	0.75	3	0.53
Fruit, No juice (cups)	Intervention	21	26	5	0.30	4	0.37
	Comparison	20	25	5	0.36	5	0.37
	Difference			0	0.96	−1	0.91
Vegetables, No fries (cups)	Intervention	44	46	2	0.81	4	0.58
	Comparison	38	50	12	0.06	12	0.07
	Difference			−11	0.25	−9	0.34
Grains, All (cups)	Intervention	29	29	0	1.00	0	0.97
	Comparison	19	23	3	0.41	3	0.45
	Difference			−3	0.58	−3	0.60
Beverages, Milk (fluid oz)	Intervention	21	25	4	0.30	6	0.10
	Comparison	24	17	−7	0.03 *	−7	0.03 *
	Difference			11	0.03 *	13	0.01 **

^a^ Results are from mixed-effects linear regression models. Adjusted models control for ethnicity (>50% black students), number of 3, 4 and 5-year olds enrolled, director education level (HS/Community College vs. Some/All 4-yr College/Graduate Degree), the number of years the center has been in operation, participation in the Child and Adult Care Food Program, and the center’s profit status (for-profit vs. nonprofit). All models (adjusted and unadjusted) include center random effects and robust standard errors. Results are reported as predicted probabilities and marginal effects. Percentages of food, beverages and nutrients are presented at the center-level, averaging across the three included children per center. ^b^ *p*-values are for change from baseline within study arm or difference-in-difference across study arms. * Significant at the *p* = 0.05 level; ** Significant at the *p* = 0.01 level.

## Supplementary Materials

The following are available online at https://www.mdpi.com/2072-6643/12/10/3024/s1, Table S1: Percent of centers serving different types of milk, pre- and post-intervention (*n* = 64 centers), Table S2: Change in absolute quantity of foods, beverages, and macro and micronutrients wasted per child per day by study arm (*n* = 64 centers, averaging across included children).
