# Supplementary material for: Preschool Healthy Food Policy Did Not Increase Percent of Food Wasted: Evidence from the Carolinas
Source: Nutrients. 2020 Oct 2;12(10):3024. doi: 10.3390/nu12103024 (PMC7600022; doi:10.3390/nu12103024)
Supplement: Supplementary file 1 [file nutrients-12-03024-s001.pdf]

S1: Percent of centers serving different types of milk, pre- and post-intervention (n=64 centers)

	Intervention (South Carolina)		Comparison (North Carolina)	
	Pre (n=34)	Post (n=33)	Pre (n=30)	Post (n=26)
Whole milk	17.6 (6)	6.1 (2)	6.7 (2)	3.8 (1)
Reduced fat	41.2 (14)	33.3 (11)	33.3 (10)	46.2 (12)
Low fat or nonfat	76.5 (26)	81.8 (27)	73.3 (22)	76.9 (20)
Nondairy milk	3.4 (1)	3.0 (1)	3.3 (1)	0.0 (0)
Ready-to-drink, flavored, whole	0.0 (0)	0.0 (0)	0.0 (0)	0.0 (0)
Ready-to-drink, flavored, reduced fat	0.0 (0)	3.0 (1)	0.0 (0)	0.0 (0)
Ready-to-drink, flavored, low/no fat	3.4 (1)	6.1 (2)	3.3 (1)	3.8 (1)

S2: Change in absolute quantity of foods, beverages, and macro and micronutrients wasted per child per day by study arm (n=64 centers, averaging across included children)

			Unadjusted[a]			Adjusted[a]	
		Baseline	Follow-Up	Diff. from Baseline	p-value[b]	Diff. from Baseline	p-value[b]
Total foods, beverages wasted (g)	Intervention	266.52	316.11	49.59	0.17	60.25	0.08
	Comparison	215.67	231.37	15.70	0.59	14.16	0.62
	Difference			33.89	0.46	46.08	0.30
Fruit, all (cups)	Intervention	0.16	0.19	0.04	0.39	0.02	0.64
	Comparison	0.18	0.25	0.08	0.09	0.07	0.09
	Difference			-0.04	0.51	-0.06	0.36
100% Fruit Juice (fluid oz)	Intervention	0.35	0.38	0.03	0.89	0.01	0.95
	Comparison	0.43	0.88	0.45	0.12	0.45	0.11
	Difference			-0.42	0.22	-0.44	0.21
Fruit, No juice (cups)	Intervention	0.11	0.15	0.03	0.32	0.02	0.58
	Comparison	0.12	0.14	0.02	0.62	0.01	0.68
	Difference			0.02	0.73	0.004	0.94
Vegetables, All (cups)	Intervention	0.22	0.34	0.12	0.01**	0.13	0.01**
	Comparison	0.13	0.24	0.10	0.01**	0.10	0.01**
	Difference			0.02	0.78	0.03	0.68
French fries (cups)	Intervention	0.01	0.001	-0.01	0.28	-0.01	0.28
	Comparison	0.002	0.00	0.002	0.29	0.001	0.37
	Difference			-0.01	0.35	-0.01	0.34

			Unadjusted[a]			Adjusted[a]	
		Baseline	Follow-Up	Diff. from Baseline	p-value[b]	Diff. from Baseline	p-value[b]
Vegetables, No fries (cups)	Intervention	0.21	0.34	0.13	0.003**	0.14	0.001**
	Comparison	0.13	0.24	0.11	0.01*	0.10	0.01**
	Difference			0.03	0.64	0.04	0.54
Grains, All (cups)	Intervention	0.36	0.50	0.15	0.06	0.16	0.05*
	Comparison	0.31	0.37	0.06	0.46	0.06	0.49
	Difference			0.09	0.44	0.10	0.37
Grains, Whole (cups)	Intervention	0.004	0.01	0.01	0.59	0.01	0.56
	Comparison	0.01	0.02	0.01	0.77	0.01	0.78
	Difference			0.00	0.99	0.00	0.98
Meat, fish, poultry, eggs, nuts, seeds (oz)	Intervention	0.33	0.45	0.11	0.34	0.12	0.37
	Comparison	0.32	0.32	-0.01	0.96	-0.01	0.93
	Difference			0.12	0.53	0.13	0.51
Yogurt (cups)	Intervention	0.01	0.00	-0.01	0.17	-0.01	0.17
	Comparison	0.00	0.003	0.003	0.31	0.003	0.30
	Difference			-0.01	0.10	-0.01	0.10
Cheese (oz)	Intervention	0.12	0.06	-0.06	0.34	-0.06	0.36
	Comparison	0.06	0.05	-0.01	0.87	-0.01	0.85
	Difference			-0.05	0.48	-0.05	0.51
Fats, cream (tablespoons)	Intervention	0.002	0.001	0.001	0.39	0.001	0.40
	Comparison	0.00	0.002	0.002	0.31	0.002	0.31
	Difference			0.003	0.20	0.003	0.19
Fats, butter, oil, margarine (teaspoon)	Intervention	0.18	0.32	0.14	0.15	0.15	0.13
	Comparison	0.08	0.23	0.15	0.08	0.14	0.10
	Difference			-0.01	0.94	0.01	0.93
Sweets, Candy (grams)	Intervention	0.00	0.00	0.00		0.002	0.42
	Comparison	0.00	0.20	0.20		0.20	0.15
	Difference			-0.20		-0.20	0.15
Sweets, Frosting (tablespoons)	Intervention	0.004	0.02	0.01	0.22	0.02	0.21
	Comparison	0.04	0.05	0.01	0.76	0.01	0.73
	Difference			0.01	0.89	0.01	0.89
Beverages, All (fluid oz)	Intervention	5.33	5.56	0.23	0.81	0.56	0.56
	Comparison	4.54	4.59	0.05	0.96	0.04	0.97
	Difference			0.18	0.89	0.52	0.68
Beverages, Milk (fluid oz)	Intervention	3.35	3.98	0.63	0.37	1.05	0.09
	Comparison	2.95	2.61	-0.35	0.46	-0.36	0.43
	Difference			0.97	0.24	1.41	0.07

		Unadjusted[a]				Adjusted[a]	
		Baseline	Follow-Up	Diff. from Baseline	p-value[b]	Diff. from Baseline	p-value[b]
Beverages, No milk (fluid oz)	Intervention	1.28	0.83	-0.44	0.35	-0.50	0.31
	Comparison	0.73	0.19	-0.54	0.11	-0.53	0.13
	Difference			0.10	0.87	0.03	0.96
Sugar-sweetened beverages (fluid oz)	Intervention	0.33	0.00	-0.33	0.26	-0.35	0.26
	Comparison	0.07	0.00	-0.07	0.31	-0.07	0.29
	Difference			-0.27	0.38	-0.28	0.38
Water (fl oz)	Intervention	0.94	0.83	-0.11	0.77	-0.16	0.69
	Comparison	0.66	0.21	-0.46	0.14	-0.44	0.16
	Difference			0.35	0.47	0.29	0.57
Macronutrients							
Calories (kcal)	Intervention	209.40	264.50	55.10	0.05*	62.69	0.02*
	Comparison	180.81	209.76	28.96	0.31	27.04	0.34
	Difference			26.15	0.52	35.65	0.37
Total Fat (g)	Intervention	6.49	7.51	1.01	0.35	1.21	0.28
	Comparison	5.77	5.38	-0.39	0.77	-0.44	0.73
	Difference			1.40	0.41	1.65	0.34
Total Carbohydrates (g)	Intervention	29.15	38.60	9.45	0.04*	10.29	0.02*
	Comparison	25.70	33.13	7.42	0.06	7.12	0.07
	Difference			2.02	0.74	3.17	0.59
Total Protein (g)	Intervention	9.52	11.95	2.42	0.07	2.95	0.02*
	Comparison	7.70	8.11	0.41	0.77	0.32	0.82
	Difference			2.02	0.30	2.63	0.16
Animal Protein (g)	Intervention	6.59	7.79	1.19	0.27	1.75	0.09
	Comparison	5.46	4.94	-0.51	0.65	-0.53	0.62
	Difference			1.70	0.27	2.29	0.13
Vegetable Protein (g)	Intervention	2.93	4.17	1.24	0.04*	1.20	0.03*
	Comparison	2.29	3.12	0.83	0.10	0.78	0.13
	Difference			0.41	0.60	0.42	0.59
Micronutrients							
Total sugar (g)	Intervention	14.12	17.80	3.68	0.10	4.32	0.04*
	Comparison	13.74	16.80	3.06	0.15	2.94	0.16
	Difference			0.63	0.84	1.38	0.64
Sodium (mg)	Intervention	360.62	487.87	127.25	0.07	135.30	0.05*
	Comparison	291.37	347.32	55.95	0.28	53.26	0.31
	Difference			71.30	0.41	82.04	0.35
Iron (mg)	Intervention	1.74	2.35	0.61	0.12	0.61	0.13
	Comparison	1.44	1.64	0.20	0.54	0.18	0.57
	Difference			0.41	0.42	0.43	0.40
Folate (mcg)	Intervention	54.17	76.04	21.87	0.11	24.52	0.07
	Comparison	39.59	47.38	7.79	0.36	6.84	0.41
	Difference			14.08	0.38	17.68	0.28
Zinc (mg)	Intervention	1.33	1.86	0.53	0.06	0.49	0.05*

		Baseline	Follow-Up	Unadjusted[a] Diff. from Baseline	p-value[b]	Adjusted[a] Diff. from Baseline	p-value[b]
	Comparison	1.24	1.17	-0.07	0.80	-0.09	0.77
	Difference			0.60	0.14	0.58	0.14
Vitamin A (IU)	Intervention	1911.61	3900.68	1989.07	0.10	2113.53	0.09
	Comparison	1253.84	2155.41	901.57	0.18	898.39	0.18
	Difference			1087.50	0.43	1215.14	0.39
Vitamin C (mg)	Intervention	11.70	15.89	4.19	0.21	4.33	0.19
	Comparison	6.50	9.15	2.65	0.30	2.47	0.32
	Difference			1.54	0.71	1.87	0.65
Vitamin D (mcg)	Intervention	1.41	1.69	0.29	0.30	0.46	0.06
	Comparison	1.23	1.07	-0.15	0.38	-0.16	0.35
	Difference			0.44	0.18	0.62	0.04*
Vitamin E (mg)	Intervention	0.83	1.08	0.25	0.28	0.26	0.27
	Comparison	0.54	0.67	0.13	0.24	0.13	0.24
	Difference			0.11	0.66	0.13	0.62

[a] Results are from linear mixed-effects regression models. Adjusted models control for ethnicity (>50% black students), number of 3, 4 and 5-year olds enrolled, director education level (HS/Community College vs. Some/All 4-yr College/Graduate Degree), the number of years the center has been in operation, participation in the Child and Adult Care Food Program, and the center's profit status (for profit vs. nonprofit). All models (adjusted and unadjusted) account for clustering within centers by clustering standard errors at the center-level. Results are reported as predicted probabilities and marginal effects. Quantities of food, beverages and nutrients are presented at the center-level, averaging across the three included children per center.

[b] P-values are for change from baseline within study arm or difference-in-difference across study arms.

*Significant at the p = 0.05 level

**Significant at the p = 0.01 level
